# Deep cervical lymphaticovenous anastomosis for Alzheimer’s disease: theoretical foundations, regulatory suspension, and translational challenges

**DOI:** 10.3389/fragi.2026.1849207

**Published:** 2026-06-02

**Authors:** Gang Li, Shusheng Jiao, Yi Zhou, Xiaofang Cheng

**Affiliations:** Department of Neurology, Bethune International Peace Hospital, Shijiazhuang, China

**Keywords:** Alzheimer’s disease, cerebrospinal fluid drainage, clinical safety, DCLVA, deep cervical lymphaticovenous anastomosis, glymphatic system, meningeal lymphatic vessels, translational medicine

## Abstract

Alzheimer’s disease (AD) is a neurodegenerative disorder characterized by pathological changes in β-amyloid protein deposition, abnormal tau protein phosphorylation neurofibrillary tangles, and chronic neuroinflammation. Recent studies have shown that the glymphatic-meningeal-cervical lymphatic system pathway plays a crucial role in the clearance of intracranial metabolic waste. Dysfunction of this system may lead to a decrease in the clearance efficiency of Aβ and tau proteins. Deep cervical lymphaticovenous anastomosis (DCLVA) has been proposed as a novel surgical approach to enhance cervical lymphatic drainage, reduce Aβ/tau accumulation, and improve cognitive function in patients with AD. However, on 8 July 2025, the National Health Commission of China issued a notice prohibiting the clinical application of “deep cervical lymphaticovenous anastomosis” for the treatment of AD. This article provides a narrative review with critical appraisal of the theoretical basis, surgical mechanisms, and clinical evidence of DCLVA for AD. We objectively evaluate the strengths and limitations of current clinical studies, critically appraise the uncertainty of underlying physiology, and comprehensively analyze the potential risks, safety concerns, and translational obstacles that led to regulatory suspension. We further clarify unresolved scientific questions including pressure gradients, lymphatic contractility, reflux risk, anastomotic patency, and biomarker validation. By framing DCLVA within the context of its clinical prohibition, we provide clinicians and researchers with a balanced appraisal that acknowledges both the procedure’s potential and the substantial gaps that must be addressed before widespread application can be justified.

## 1 Introduction

On 8 July 2025, the National Health Commission of China issued an unusual notice: the application of “deep cervical lymphaticovenous anastomosis” for the treatment of Alzheimer’s disease was formally prohibited (National Health Commission of China, 2025). This regulatory action, occurring just years after the first reported cases of DCLVA in China, reflects a stark reality: despite promising theoretical foundations and preliminary reports of cognitive improvement, the procedure has outpaced the evidence supporting its safety and efficacy.

Alzheimer’s disease (AD) is the most common neurodegenerative disorder and is characterized by abnormal deposition of beta-amyloid (Aβ)-forming amyloid plaques, hyperphosphorylation of tau protein leading to neurofibrillary tangles, and impaired clearance of metabolic waste (Kamatham et al., 2024; Hu et al., 2024). Although the exact etiology of AD remains unclear, an increasing number of studies suggest that dysfunction of the glymphatic system of the brain plays a key role in the occurrence and development of AD. Under normal circumstances, the brain’s glymphatic system is responsible for removing harmful protein deposits (Wu et al., 2022; Li et al., 2020). However, with age or other factors, its dysfunction may lead to the accumulation of Aβ and tau proteins and other metabolic wastes, causing neuronal damage and apoptosis, ultimately resulting in the occurrence and progression of AD (Scheltens et al., 2021; Nedergaard and Goldman, 2020; Chong et al., 2025).

There were approximately 55 million AD and related dementia patients worldwide in 2023, and it is estimated that this number will reach 139 million by 2050, with AD accounting for 60%–70% of the total (Global, 2024; GBD 2019 Dementia Forecasting Collaborators, 2022). Currently, clinical interventions for AD mainly focus on symptomatic treatment. Drugs such as cholinesterase inhibitors and excitatory amino acid receptor inhibitors can only alleviate symptoms but cannot reverse or prevent disease progression (Kamatham et al., 2024; Passeri et al., 2022). Therefore, there is an urgent need to develop novel treatment methods.

In recent years, the discovery of the glymphatic system in the central nervous system (CNS) has provided a new perspective for AD research (Iliff et al., 2012; Louveau et al., 2015). Studies have shown that the glymphatic system plays a crucial role in the removal Aβ, tau proteins and other metabolic wastes. Its dysfunction may lead to abnormal deposition of these pathological proteins and accelerate the progression of AD (Busche and Hyman, 2020). Moreover, dysfunction of the glymphatic system may occur earlier than typical pathological changes in AD, suggesting its potential value in early diagnosis. The glymphatic system and meningeal lymphatic vessels play key roles in maintaining fluid balance in the brain, clearing metabolic wastes, and in the pathogenesis of central nervous system diseases (Li et al., 2022; Yamada and Iwatsubo, 2024). The glymphatic system maintains brain homeostasis by promoting the exchange of cerebrospinal fluid (CSF) and interstitial fluid, whereas meningeal lymphatic vessels are involved in CSF drainage and immune regulation. Dysfunction of both systems is closely related to neurodegenerative diseases such as AD. Evidence suggests that meningeal lymphatic vessels (mLVs) play an important role in the drainage of CSF and interstitial fluid (Ahn et al., 2019).

Deep cervical lymphaticovenous anastomosis (DCLVA) is a microsurgical technique aimed at enhancing the drainage of cerebrospinal fluid (CSF) and interstitial fluid and has been proposed as a potential strategy for treating AD (Ma et al., 2025). Preliminary results from exploratory studies in China suggest that DCLVA may improve cognitive function and biomarkers (Chen JY. et al., 2025), but this technique is still in the early stages of clinical research and its indications and contraindications are not yet clear. However, there is a lack of high-quality evidence to support its safety and efficacy, including unresolved physiological uncertainties (e.g., rate-limiting steps in clearance, venous-lymphatic pressure gradient, reflux risk) and inadequate outcome measurement (e.g., lack of quantified biomarkers and long-term follow-up).

This review critically examines the trajectory of DCLVA for AD-from its mechanistic rationale to its premature clinical adoption and subsequent prohibition. Rather than simply cataloging existing studies, we aim to: (i) delineate the unresolved physiological uncertainties that undermine the procedure’s theoretical foundation; (ii) systematically characterize the clinical contraindications that necessitate regulatory caution; and (iii) propose a pragmatic roadmap for future translational research. By framing DCLVA within the context of its clinical prohibition, we seek to provide clinicians and researchers with a balanced appraisal that acknowledges both the procedure’s potential and the substantial gaps that must be addressed before its widespread application can be justified.

## 2 Theoretical basis of venous anastomosis: from rationale to uncertainty

### 2.1 The association between the brain glymphatic and meningeal lymphatic system and AD

In 2012, Nedergaard’s group revealed that cerebrospinal fluid (CSF) circulates within the brain interstitial space via paravascular pathways, with astrocytic aquaporin-4 (AQP4) playing a critical role in metabolic waste clearance (Iliff et al., 2012). In 2015, Louveau et al. identified functional lymphatic vessels along the dural sinuses. These vessels display the molecular characteristics of lymphatic endothelium, mediate the drainage of CSF-derived fluid and immune cells, and are directly connected to the deep cervical lymph nodes (Louveau et al., 2015). In 2025, a study published in Cell further revealed the molecular mechanism by which the meningeal lymphatic system regulates synaptic function through the “lymphatic-microglial-IL-6 axis”, providing a new target for AD treatment (Kim et al., 2025). Preclinical studies have confirmed that blocking cervical lymphatic vessels reduces cerebral Aβ clearance and exacerbates AD-like pathology (Wang et al., 2019).

International research has further validated the role of the glymphatic and meningeal lymphatic systems in human AD. Ringstad et al. used MRI to track CSF tracer distribution in the human brain and found delayed tracer clearance in the dementia cohort, providing the first *in vivo* human evidence of impaired brain clearance function in AD (Ringstad et al., 2018). Eide et al. confirmed via multiphase MRI that sleep deprivation impairs molecular clearance in the human brain by inhibiting glymphatic function (Eide et al., 2021). Yamamoto et al. used intrathecal gadolinium contrast-enhanced MRI to verify that contrast-labeled CSF moves through perivascular spaces into the brain parenchyma, directly supporting the existence of the glymphatic pathway in humans (Yamamoto et al., 2024).

Chachaj et al. systematically summarized the clearance mechanisms of the brain glymphatic and meningeal lymphatic systems and their potential therapeutic value for AD (Chachaj et al., 2023). Abnormal immune cell activity in the meningeal lymphatic system of AD patients can increase neuroinflammation and hinder lymph flow (Fu et al., 2021), while deep cervical lymphaticovenous anastomosis (DCLVA) can not only improve lymphatic flow, but also reduce the concentration of neuroinflammatory factors and alleviate tissue hypersensitivity (Louveau et al., 2018), suggesting its dual potential in improving waste clearance and inhibiting neuroinflammation.

### 2.2 Mechanism of the surgery

The glymphatic-meningeal lymphatic clearance cascade is the core physiological basis of DCLVA. Driven by arterial pressure waves, CSF flows through the perivascular spaces of penetrating arteries, enters the brain parenchyma through highly polarized AQP4 on astrocyte foot processes, and mixes with interstitial fluid (ISF). The mixed fluid then flows toward deep veins, accumulates in venous perivascular spaces, and finally drains into cervical lymphatic vessels through dural lymphatics (Louveau et al., 2015; Aspelund et al., 2015), completing the intracranial to extracranial waste clearance process.

Yoon et al. have made landmark contributions to clarifying the CSF drainage pathway to cervical lymph nodes: basal meningeal lymphatic vessels (mLVs) are key hubs for CSF macromolecule clearance, and both mLV integrity and CSF drainage capacity decline with aging. In 2024, they found that the nasopharyngeal lymphatic plexus is a major hub for CSF outflow to deep cervical lymph nodes—this plexus has unique structural characteristics, while downstream deep cervical lymphatics have typical semilunar valves and smooth muscle coverage, whose CSF transport function is regulated by α-adrenergic and nitric oxide signaling in smooth muscle cells (Yoon et al., 2024). In 2025, their further study confirmed that CSF enters skull base meningeal initial lymphatics, flows through extracranial periorbital, olfactory, nasopharyngeal and hard palate lymphatics, and then reaches submandibular lymph nodes via smooth muscle-covered superficial cervical lymphatics; aged mice show reduced nasal and hard palate lymphatics and impaired CSF outflow to cervical lymph nodes, and non-invasive mechanical stimulation of cervical lymphatics can reverse this impairment, highlighting the importance of cervical lymphatic function for intracranial CSF clearance and providing a potential non-invasive alternative to DCLVA (Jin et al., 2025).

In AD, dysfunction of the meningeal lymphatic system leads to impaired ISF and CSF drainage, and subsequent accumulation of Aβ and other metabolic wastes, which is one of the core causes of pathological protein deposition. DCLVA is a super-microsurgical technique designed to reconstruct the impaired cervical lymphatic drainage pathway: under a 10–20× magnification microscope, 0.1–0.3 mm diameter deep cervical lymphatic vessels (or afferent vessels of deep cervical lymph nodes) are anastomosed with tributaries of the internal jugular vein (IJV). This procedure establishes a direct lymphatic-venous anastomotic channel, reconstructs the lymphatic return pathway, improves CSF and interstitial fluid drainage efficiency, promotes the excretion of Aβ and tau proteins, and restores the impaired meningeal lymphatic drainage function in AD patients, thereby alleviating AD symptoms and delaying disease progression (Xie QP. et al., 2025).

Notably, cervical lymphatic drainage is not a passive process: the driving force for CSF/lymph flow is generated by the intrinsic contractility of cervical lymphatic vessels and extrinsic pumping (e.g., arterial pulsatility, respiratory movement). In healthy individuals, intracranial pressure is only slightly higher than central venous pressure (CVP), and CVP in AD patients (mostly elderly) fluctuates significantly with posture, Valsalva maneuver and cardiopulmonary status. The pressure relationship between deep cervical lymphatics and the internal jugular vein in humans remains poorly characterized. In the supine position, the venous side typically exhibits a modestly higher pressure than the lymphatic side, creating an unfavorable gradient for lymphatic drainage. However, direct human data on cervical lymphatic pressure are extremely limited, with most pressure physiology derived from animal models and extrapolated from peripheral lymphology studies. A critical concern of DCLVA is that the surgery may resect or damage the contractile segments of deep cervical lymphatic vessels responsible for active pumping, which could potentially hinder rather than enhance CSF drainage.

**FIGURE 1:**
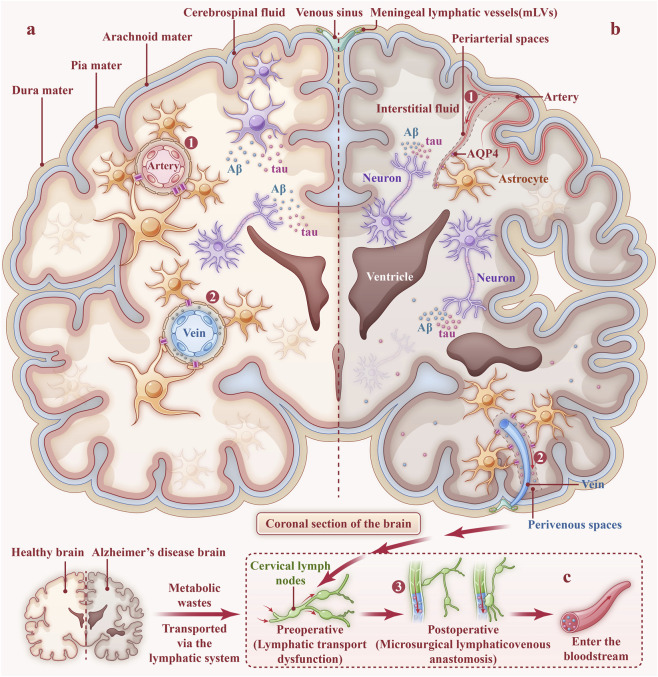
Glymphatic waste clearance under physiological and pathological conditions, and the surgical intervention strategy. **(a)** Healthy brain. Cerebrospinal fluid (CSF) enters the brain parenchyma through arterial perivascular spaces (①), exchanges with interstitial fluid (ISF) via astrocytic aquaporin-4 (AQP4), and drains metabolic wastes (e.g., Aβ, tau) via venous perivascular spaces (②) to meningeal and cervical lymphatics, ultimately entering the systemic circulation. **(b)** Alzheimer’s disease (AD) brain. Impaired CSF-ISF exchange, caused by perivascular dilation and loss of AQP4 polarization, leads to massive accumulation of Aβ and tau in the brain parenchyma (②), accelerating neurodegeneration. **(c)** Deep cervical lymphaticovenous anastomosis (DCLVA). This procedure bypasses dysfunctional cervical lymphatic pathways (③), diverting waste-laden lymph directly into the venous system to promote peripheral clearance of cerebral toxic metabolites.

Sustained clinical efficacy of DCLVA depends on two core physiological conditions: a stable favorable pressure gradient across the anastomosis (lymphatic pressure > venous pressure) and effective prevention of venous-to-lymphatic reflux-a major failure mode of all lymphaticovenous anastomosis surgeries. The pathways for the drainage of metabolic waste in the human brain and the schematic diagram of the DCLVA surgery for AD are shown in Figure 1.

## 3 Introduction to the surgery: experience accumulation and standardization gaps

### 3.1 Surgical procedure

DCLVA is a super-microsurgical procedure that requires anastomosis of deep cervical lymphatic vessels or lymph nodes with the internal jugular vein under a high-power microscope (10–20 times magnification) to allow lymph to enter the circulatory system and relieve lymphatic obstruction (Onoda et al., 2023). These lymphatic vessels and lymph nodes are very fine, with some as small as 0.1 mm. The surgical process is delicate and complex, requiring the surgeon to have superb microsurgical skills. The operation usually lasts approximately 3–6 h, and during the operation, damage to the surrounding blood vessels and nerves must be avoided (Lu et al., 2022). In some cases, enlarged and abnormal lymph nodes must be removed. Potential perioperative risks of DCLVA include: vascular injury (IJV or cervical artery), nerve palsy (cranial nerves IX–XII), infection, lymphatic leakage, thrombosis of the anastomosis, and venous thromboembolism; long-term risks include anastomosis occlusion, venous-to-lymphatic reflux, and persistent lymphatic dysfunction.

### 3.2 Indications and evaluation for surgery

Not all patients with AD are suitable candidates for DCLVA surgery. Currently, it is believed that patients with moderate to severe AD can tolerate surgery, especially those who have poor responses to drug treatment. Patients with early-stage mild disease should prioritize drug and non-drug treatments. In this regard, current pharmacotherapies for early-stage AD, including the anti-amyloid monoclonal antibodies lecanemab and donanemab, have demonstrated efficacy in slowing cognitive decline in randomized controlled trials and represent the current standard of care. Any future evaluation of DCLVA must therefore compare the procedure against these established treatments rather than against placebo or no treatment. Before surgery, a comprehensive assessment should be conducted through cognitive tests (such as Mental State Examination Scale (MMSE) and MOCA scales), imaging examinations (such as PET-CT), and cerebrospinal fluid analysis, and a personalized plan should be developed by a multidisciplinary team (Xie QP. et al., 2025). Preoperative evaluation should also include assessment of cervical lymphatic flow (e.g., lymphoscintigraphy) and central venous pressure to predict anastomosis patency and pressure gradient.

## 4 Clinical application outcomes: a critical appraisal

Since Lu and Xie et al. first introduced DCLVA for cognitive impairment at Hangzhou Qiu Shi Hospital in 2018 and reported an initial case under 3D microscopy (Lu et al., 2022), several clinical case series and observational studies of DCLVA for AD have been conducted in China. Xie et al. reported significant improvements in cognitive function (MMSE/MOCA) and behavioral symptoms in 50 patients over a 9-month follow-up period (Xie et al., 2024). Chen et al. performed DCLVA in 26 AD patients and observed that approximately 60% of caregivers reported symptomatic improvement at 1 month postoperatively, accompanied by a significant increase in MMSE scores (Chen JY. et al., 2025). A 2024 study further suggested better outcomes in the DCLVA-plus-drug group compared with the drug-only group after 24 months of follow-up Fu, (2024).

Critical appraisal of these findings reveals major limitations that preclude definitive clinical conclusions. All available evidence is derived from small-sample, single-center, uncontrolled observational studies without randomization, blinding, or control groups. Outcome assessment is largely restricted to subjective cognitive scales (MMSE, MOCA) and caregiver-reported symptomatic changes, rather than objective, validated biomarkers such as brain Aβ/tau PET or cerebrospinal fluid measures. No study has confirmed improvements in daily functioning (ADL/IADL), long-term disease modification, or reduced neurodegeneration. Furthermore, no clinical trial has verified anastomotic patency, flow direction, pressure gradients, or the actual enhancement of glymphatic clearance after surgery.

In summary, while preliminary data suggest that DCLVA may correlate with short-term subjective cognitive improvement in selected patients, the overall quality of evidence is low and insufficient to confirm efficacy or clinical utility. A summary of evidence levels for DCLVA in AD treatment is provided in Table 1.

**TABLE 1:** Level of evidence for deep cervical lymphaticovenous anastomosis (DCLVA) in Alzheimer’s disease treatment.

Aspect	Evidence level	Study type	Key limitations
Theoretical basis	Level 2 (Moderate)	Animal studies + Human anatomy	Unresolved human physiological uncertainties (pressure, rate-limiting steps)
Short-term cognitive effect	Level 4 (Low)	Small-sample, uncontrolled cohort	Only MMSE/MOCA, no quantified biomarkers/functional outcomes
Surgical safety	Level 4 (Low)	Retrospective case series	No structured adverse event reporting, no long-term follow-up for anastomosis failure
Mechanistic validation	Level 5 (Very Low)	No human studies	No verification of anastomosis patency/flow direction, no glymphatic function assessment

## 5 Discussion and conclusion

### 5.1 Discussion

The National Health Commission’s prohibition of DCLVA for AD was not based on evidence of harm, but rather on the absence of evidence for benefit and the potential for premature dissemination. DCLVA procedure creates a direct pathway for deep lymphatic vessels and veins in the neck, aiming to enhance the lymphatic return from the brain to the neck, accelerate the excretion of metabolic wastes such as Aβ, and optimize the microenvironment within the brain. Despite its strong theoretical rationale, encouraging preliminary clinical observations and attenuate cognitive dysfunction and biomarker abnormalities in severe Alzheimer’s disease (Fu et al., 2026), this review critically demonstrates that DCLVA remains an experimental intervention with unresolved physiological, methodological, and safety concerns.

First, we have positioned DCLVA relative to approved anti-amyloid therapies (lecanemab, donanemab), which represent the current standard of care for early AD by targeting Aβ clearance. DCLVA acts via a distinct glymphatic/lymphatic pathway and is not a replacement but a complementary strategy under investigation. However, the efficacy evaluation of DCLVA surgery for AD mainly involves comprehensive judgment through cognitive function scales (such as MMSE and MOCA), imaging examinations (such as observing changes in protein deposits in the brain with head PET-CT), and improvements in patients’ daily living abilities and mental and behavioral symptoms (Jack et al., 2024), rather than objective biomarkers such as brain Aβ/tau PET or cerebrospinal fluid measures. Most clinical studies are small-sample, uncontrolled, and single-center, lacking high-level evidence from randomized controlled trials (RCTs) and long-term follow-up. All clinical data are from Chinese populations, differences in cervical lymphatic anatomy, APOE4 prevalence, and comorbidity profiles may limit extrapolation to Western cohorts. Short-term symptomatic improvement does not equate to disease modification, and symptom rebound in some patients suggests potential placebo effects rather than genuine structural or functional recovery (Tu et al., 2025). The placebo effect is substantial in surgical trials using subjective cognitive scales (MMSE/MoCA) and caregiver reports; unblinded, non-sham design limits interpretation of reported improvements. Additionally, there are limitations in detection methods, such as the DTI-ALPS index (Diffusion Tensor Image Analysis ALong the Perivascular Space), the DTI-ALPS index is a semi-quantitative MRI measure used to estimate glymphatic function, which has not been fully verified for its pathophysiological significance (Ringstad, 2024; Taoka et al., 2024; Huang et al., 2024).

Second, non-invasive alternatives exist: Jin et al. demonstrated that non-invasive mechanical cervical lymphatic stimulation can enhance CSF drainage without surgery, providing a safer translational alternative to DCLVA (Jin et al., 2025). Importantly, critical physiological uncertainties remain unsolved for DCLVA. The rate-limiting step of brain waste clearance is still unclear; if the bottleneck lies in intracranial glymphatic dysfunction (e.g., impaired AQP4 polarization, reduced arterial pulsatility) rather than cervical lymphatic drainage, then DCLVA may provide limited clinical benefit. Moreover, the pressure gradient between lymphatic vessels and veins, the risk of venous reflux, and the impact of surgery on native lymphatic contractility are poorly understood, all of which may undermine long-term anastomotic patency and therapeutic efficacy. The glymphatic system’s mechanism (bulk flow vs. diffusion) and AQP4’s role remain debated in humans, leading to uncertainty in the upstream pathway targeted by DCLVA. Aβ clearance is a multi-pathway process (BBB transport, enzymatic degradation, lymphatic drainage), so DCLVA may only have a modest effect size.

Third, ethical considerations deserve explicit attention. Informed consent in moderate-severe AD patients is compromised by cognitive impairment, the risk-benefit balance of a 4-h microsurgical procedure in elderly, comorbid patients requires strict ethical review. Current evidence is insufficient to confirm the safety profile of DCLVA. Perioperative risks include vascular injury, cranial nerve damage, infection, lymphatic leakage, and thromboembolism. Surgical inflammatory response include cervical DCLVA induces local and systemic inflammation, which may paradoxically exacerbate neuroinflammation and glymphatic dysfunction in AD, this potential harm requires further investigation. Long-term risks include anastomotic occlusion, venous reflux, and persistent lymphatic dysfunction. The lack of standardized surgical protocols, inclusion criteria, and outcome measures further hinders clinical translation and cross-study comparison.

In summary, this critical review confirms that DCLVA is not yet ready for routine clinical application in AD, which supports the clinical prohibition issued by the National Health Commission of China. Future research must prioritize rigorous RCTs, objective biomarker validation, long-term safety monitoring, and physiological mechanism clarification before any clinical re-introduction can be considered.

### 5.2 Conclusion

DCLVA represents a biologically plausible strategy targeting the glymphatic-meningeal-cervical lymphatic system in AD, but it remains at an early investigational stage. Summary of published clinical studies of DCLVA for AD and key limitations are shown in Table 2. Current evidence is insufficient to support its clinical efficacy or safety, and critical physiological and methodological challenges persist.

**TABLE 2:** Summary of published clinical studies of DCLVA for AD.

First author	N	Follow-up	Main outcomes	Key limitations
Lu et al. (2022)	1	9 months	Cognitive improvement	Single case
Xie et al. (2024)	50	9 months	MMSE/MoCA improved	Uncontrolled, subjective
Fu, (2024)	–	24 months	Better than drug alone	Uncontrolled, conference abstract
Li et al. (2024)	1	5 weeks	Cognitive improvement	Single case
Chen et al. (2025a)	26	1 month	60% caregiver-reported improvement	Uncontrolled, short follow-up
Li et al. (2025)	7	1 month	Promote the clearance of pathological proteins, reduce intracranial Aβ protein levels, and improve clinical symptoms	Single-Arm Observational Study
Gan et al. (2025)	4	1 month	MMSE increased	Uncontrolled, short follow-up
Chen et al. (2025b)	1	3 months	MMSE increased	Single case
Xie et al. (2025b)	1	4 months	MMSE/MoCA improved and reduction in abnormal brain amyloid deposits	Single case
Tang et al. (2025)	28	1 week	64.3% MMSE improvement; tau detected in lymph nodes	Uncontrolled, short follow-up
Fu et al. (2026)	139	6 months	MMSE elevated and observable changes across functional status and neuropsychiatric symptoms. Biomarker analyses revealed decreased cerebrospinal fluid (CSF) β-amyloid (Aβ) 42, Aβ40, and p-Tau levels postoperatively	A prospective single-arm study
Hu et al. (2026)	30	6 months	Elevated peripheral blood amyloid-β (Aβ)42 levels. The combination of Aβ42 and Aβ42/40 achieved the highest AUC (0.737) at 180 days post-surgery	Single-arm study

High-quality, multi-center, randomized controlled trials with long-term follow-up are mandatory to validate efficacy, confirm safety, clarify mechanisms, and standardize surgical and evaluative protocols. These trials should use sham surgery controls, blinded outcome assessments, and objective biomarkers as primary endpoints, rather than subjective cognitive scales alone. They should also include diverse patient populations and compare DCLVA against current standard-of-care pharmacotherapies (anti-amyloid antibodies).

This review provides clinical guidance by emphasizing that DCLVA should be restricted to ethically approved, controlled research settings rather than routine clinical practice. Only with robust scientific and clinical evidence can lymphatic-targeted surgical strategies advance toward meaningful translation for patients with AD.
